# Patients Present Earlier and Survival Has Improved, but Pre-ART Attrition Is High in a Six-Year HIV Cohort Data from Ethiopia

**DOI:** 10.1371/journal.pone.0013268

**Published:** 2010-10-11

**Authors:** Zewdie Mulissa, Degu Jerene, Bernt Lindtjørn

**Affiliations:** 1 Arba Minch Hospital, Arba Minch, Ethiopia; 2 WHO Country Office, Addis Ababa, Ethiopia; 3 Centre for International Health, University of Bergen, Bergen, Norway; University of Cape Town, South Africa

## Abstract

**Background:**

Previous studies showed higher early mortality rates among patients treated with antiretroviral drugs in settings with limited resources. One of the reasons was late presentation of patients to care. With improved access to HIV services, we expect improvements in disease stage at presentation. Our objective was to assess the effect of improved availability of HIV services on patient presentation to care and subsequent pre-ART and on-ART outcomes.

**Methodology and Principal Findings:**

At Arba Minch Hospital in Ethiopia, we reviewed baseline characteristics and outcomes of 2191 adult HIV patients. Nearly a half were in WHO stage III at presentation. About two-thirds of the patients (1428) started ART. Patients enrolled in the early phase (OR = 4.03, 95% CI 3.07–5.27), men (OR = 1.78, 95%CI 1.47–2.16), and those aged 45 years and above (OR = 2.04, 95%CI 1.48–2.82) were at higher risk of being in advanced clinical stage at presentation. The pre-treatment mortality rate was 13.1 per 100 PYO, ranging from 1.4 in the rapid scale-up phase to 25.9 per 100 PYO in the early phase. A quarter of the patients were lost to follow-up before starting treatment. Being in less advanced stage (HR = 1.9, 95% CI = 1.6, 2.2), being in the recent cohort (HR = 2.0, 95% CI = 1.6, 2.6), and rural residence (HR = 1.8, 95% CI = 1.5, 2.2) were independent predictors of pre-ART loss to follow-up. Of those who started ART, 13.4% were lost to follow-up and 15.4% died. The survival improved during the study. Patients with advanced disease, men and older people had higher death rates.

**Conclusions and Significance:**

Patients started to present at earlier stages of their illness and death has decreased among adult HIV patients visiting Arba Minch Hospital. However, many patients were lost from pre-treatment follow-up. Early treatment start contributed to improved survival. Both pre-ART and on-ART patient retention mechanisms should be strengthened.

## Introduction

Early diagnosis, timely initiation of treatment, and retention in care depend both on patient characteristics and health systems factors. [1], [2] During the early years of introduction of antiretroviral therapy (ART) in resource-limited settings, late presentation was inevitable because of lack of access to antiretroviral drugs (ARVs). In recent years, however, access to ARVs has improved and lack of drugs cannot be taken as reasons for late initiation of treatment. [3]

The Ethiopian Ministry of Health (MOH) introduced ART in 2003 on subsidized, fee-based scheme, and ART became freely available since 2005. Further, ART was decentralized to health centres in 2006, which marked the rapid scale-up phase in the history of the Ethiopian ART programme. [4] In addition to adopting the WHO-recommended public health approach, Ethiopia used innovative models such as a nationwide campaign to achieve national targets both for ART and HIV testing and counselling. [5] Whether this improved availability of HIV services has been accompanied by earlier presentation of patients to care has not been studied.

Not all patients who present at earlier stage of their illness are eligible for ART. Even when they are eligible for ART, prompt initiation of treatment will depend on several factors including availability of medicines and trained health workers. It is therefore likely that in settings with high disease burden and limited resources, some patients will either default from treatment or will even die before they are started on ART. Such pre-ART patient outcomes including death and loss rates are not adequately documented, as most of the literature has focused on outcomes after ART initiation. [6]

Here, we present data on stage shifting and pre-ART outcomes from a district hospital in southern Ethiopia. Our objective was to assess whether there had been a shift in disease stage at presentation and determine pre-ART and on-ART patient outcomes among patients enrolled in care over a six-year period.

## Methods

### Participants

We did this study at Arba Minch Hospital, located 500 Km south of Addis Ababa. The hospital started providing ART in August 2003 with financial support from the Norwegian Lutheran Mission. When Ethiopia launched the ‘free’ ART programme in 2005, the hospital became part of the national scheme for ARV delivery. Since 2005, the MOH supplied ARVs and other HIV related commodities. In close coordination with the routine work and with technical support from the University of Bergen, Norway, we set up an HIV research cohort described in detail elsewhere. [7], [8], [9] Since mid-2006, the routine HIV work at the hospital has received additional technical support from the President's Emergency Plan for AIDS Relief (PEPFAR) through Johns Hopkins University, Technical Support for the Ethiopian HIV/AIDS ART Initiative (JHU-TSEHAI).

### Description of Procedures

Patients received care according to the national HIV treatment guidelines [10], [11]. We enrolled all HIV infected patients who visited the HIV clinic on first-come first-served basis. A trained health worker did initial evaluation and subsequent follow-up of patients. Until CD4 testing was available in mid-2006, we had used only clinical and total lymphocyte count (TLC) criteria for starting treatment and follow-up.

During the early phase of ART in Ethiopia, generic combinations of stavudine (d4T), lamivudine (3TC), nevirapine (NVP), zidovudine (ZDV/AZT), and Efavirenz (EFV) were approved for use[9]. Later, Tenofovir (TDF) was added to the arsenal of first line drugs [10]. Second-line drugs included Didanosine (DDI), Abacavir (ABC), TDF, and ZDV (if not used as first line regimen), Ritonavir (RTV) and Lopinavir (LPV/r) [10].

Stavudine was given as 40 mg or 30 mg (depending on body weight) twice daily until the last 3 months (from the study closure) when the 40 mg became obsolete because of an increased toxicity[11]; 3TC as 150 mg twice daily; and NVP 200 mg daily for the first two weeks and then twice daily after that. ZDV was given at a dose of 300 mg twice daily. The dose of EFV was 600 mg daily. TDF was given as 300 mg daily, ABC as 300 mg twice daily and DDI as 250 mg or 400 mg (depending on body weight) daily. Ritonavir (RTV) was used to “boost” LPV/r with a dose of 100 mg/day; LPV/r was given as 3 capsules (400 mg LPV/r and 100 mg RTV) twice daily. All drugs were given orally.

Two data clerks maintained both paper-based and electronic records of patient information. Using a data abstraction form as a guide, we recorded date of HIV testing, date of pre-ART enrolment, WHO clinical stage, CD4 count, total lymphocyte count (TLC), history of tuberculosis, pregnancy, age, sex, and place of residence for all patients directly into an SPSS data file. To ensure the inclusion of all relevant information in the database, we did thorough cleaning of the data, cross-checked with the paper records and included additional relevant variables between April and December, 2009. One of the investigators (ZM) supervised the cohort updating.

We defined pre-ART patient outcome as: (a) ‘still under pre-ART care’-if patient was registered with the ART clinic of the hospital, had regular follow-up with the clinic and was not having follow-up at another health facility; (b) ‘lost to follow-up, ‘-if patient did not have follow-up visit at least 30 days after the last date of the next clinic appointment; (c) ‘put on ART’-if patient was started on ART in the hospital clinic; (d), ‘died before starting ART’-if patient was known to be dead as reported by treating clinicians or community health agents; and (e), ‘transferred out’-if patient moved to another health facility with confirmed written documentation of transfer out.

In patients who were started on ART, we defined patients as lost to follow up if they did not attend the hospital within the previous 30 days. For lost to follow up patients we did an ‘extended follow up’ in 2009. ‘Extended follow up’ involved home visit or phone call using community health agents. The community health agents had completed high school education and received extra training on HIV/AIDS. After each visit, they reported the status of each patient to the data clerks.

We defined the patient status after extended follow-up as: ‘Died’: if a family member, neighbour or community leader reported death of the patient. ‘Under follow up at another health facility’: if the patient was on treatment at any health facility in the region as reported by family, neighbours or community leaders. ‘Stopped treatment but alive’: if patient did not take ARVs for more than 1 month and the patient was alive and did not get ART elsewhere. ‘On traditional treatment’: if the patient reported that he or she used traditional medicines instead of ART. ‘Left the region’: if patient left the region as reported by family, neighbours or community leaders. Unknown (‘true loss’): if no information was available about patient.

### Ethics

In this study, we included all adult [age greater than 14 yrs], treatment-naïve patients enrolled in the cohort from January 2003 through 31 December, 2008. The AMH HIV cohort was ethically cleared by the National Ethics Review Committee in Ethiopia. Patients gave informed written consent for HIV testing. Some of these patients were included in previously published prospective studies for which informed written consent was obtained. [7], [8], [9] For the additional retrospective data included in this updated cohort, the study protocol was approved by the Ethics Committee of Gamo Goffa Zone Health Department. Since this study was based on retrospective data, informed consent was not feasible. To ensure confidentiality, we excluded patient identifiers from the final database. All patients received the standard care at the hospital. [10], [11]

### Statistical methods

We used SPSS version 16 for data entry and analysis. In this analysis, we stratified the patient enrolment period into three phases: (i) January 2003-August 2006 (Early phase); (ii) September 2006-August 2007 (Rapid scale-up phase); and (iii) September 2007-December 2008 (Recent phase). We used this categorization based on the chronology of Ethiopia's ART scale-up [4] We then compared the proportion of patients in each WHO clinical stage for the three phases of enrolment using Chi-square for trend test. Similarly, we compared the differences in time from HIV testing to pre-ART enrolment (in days), and time from pre-ART enrolment to ART initiation (in days).

To find out the risk factors for presenting at advanced disease stage, we used the logistic regression method. In the logistic regression analysis, we used WHO clinical stage dichotomized into advanced (Stages III and IV combined) vs. less advanced (Stages I&II combined) stages as the main outcome variable. Since CD4 testing was not available during the early phase, we did not use CD4 count cut-off points in this analysis. We included age, marital status, sex, and phase of enrolment in the final model, and reported the results as odds ratio (OR) with 95% confidence interval (CI).

Then we estimated time to death and loss to follow-up using Kaplan-Meier and Cox regression methods. We then calculated the mortality and loss to follow up rates by dividing the total number of deaths and losses by the person-year of observation (PYO) for the three time periods mentioned above.

## Results

### Baseline results

**Figure 1:**
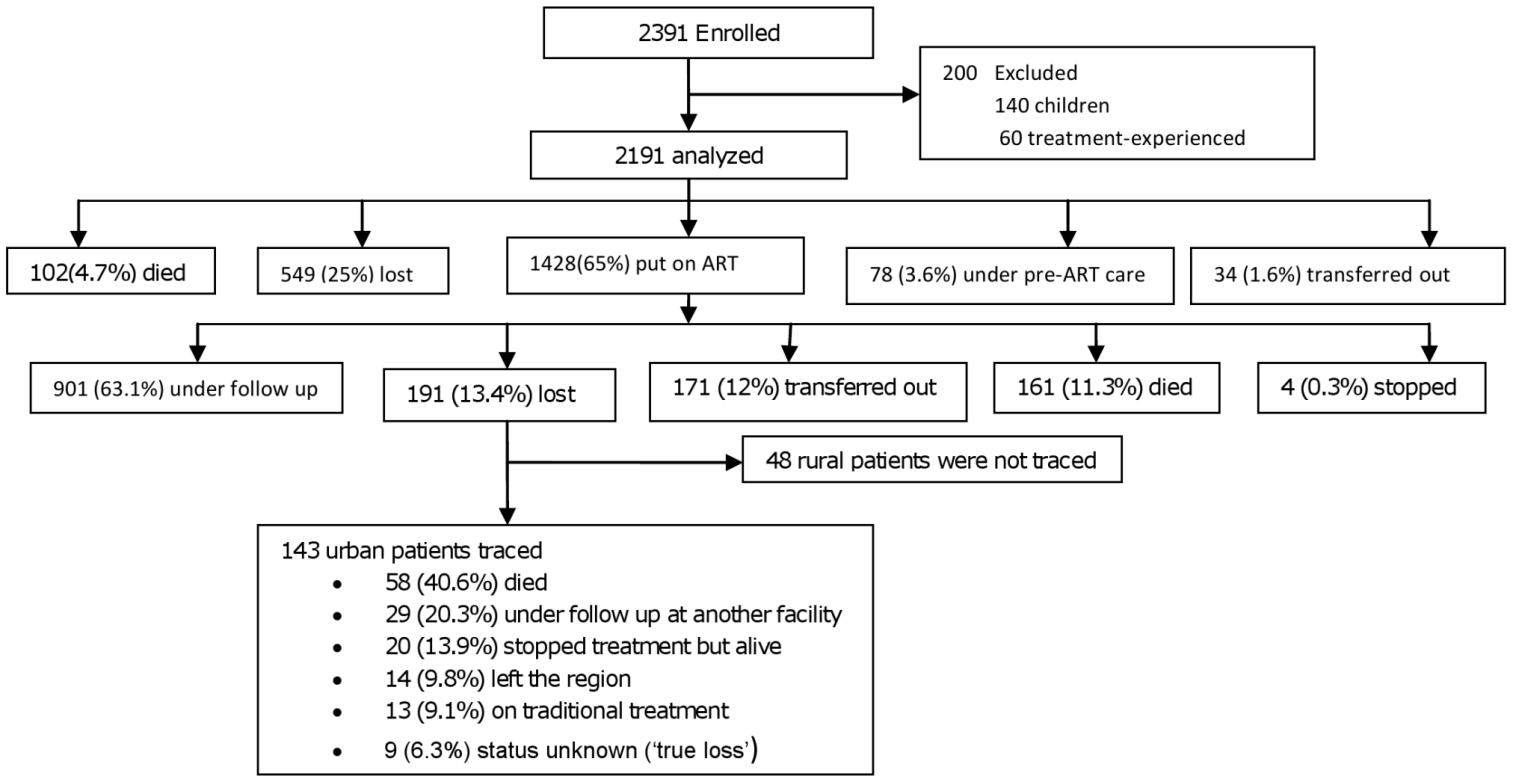
Cohort profile, Arba Minch Hospital, 2010. Of 2391 patients enrolled for care, 2191 were eligible for analysis. At the end of the pre-ART follow-up period, 25% were not in care.

Between January 2003 and 31 December 2008, we recruited and followed 2391 patients. After excluding children and treatment-experienced adults, 2191 patients were eligible for analysis (see Figure 1). Their median age was 33 years (IQR, 26–38), 56% were women, and 83% urban residents. Ninety (7.3%) of the women were pregnant at presentation. Table 1 describes the baseline characteristics of the cohort. Previous and current history of tuberculosis was reported by 11.8% and 14.9% of the patients respectively.

**Table 1:** Baseline characteristics of all treatment-naïve adult patients at enrollment, Arba Minch Hospital, 2010.

Characteristic	Number (%)
Age (yrs)	
15–24	304 (13.9)
25–34	971 (44.3)
35–44	647 (29.5)
45+	269 (12.3)
Sex	
Male	964 (44)
Female	1227 (56)
Place of residence	
Urban	1821(83.1)
Rural	370 (16.9)
Education	
Below primary education	640 (28.2)
Primary education	711 (32.5)
Secondary education and above	832 (38.0)
Missing	8 (0.4)
Marital status	
Married	1165 (53.2)
Unmarried	341 (15.6)
Widowed/er	282 (12.9)
Separated	257 (11.7)
Missing	4 (0.2)
WHO clinical stage	
I	398 (18.2)
II	424 (19.4)
III	1077 (49.2)
IV	292 (13.3)
Period of enrollment	
Early phase	509 (23.2)
Rapid scale-up phase	1015 (46.3)
Recent phase	667 (30.4)

The median time between HIV diagnosis and pre-ART enrolment was 1 day (IQR, 0–4 days) with 49% of the patients being enrolled within same day of testing. The median time from pre-ART enrolment to ART initiation was 16 days (IQR, 5–101). When stratified by year of enrolment, there was significant decline in the duration of pre-ART waiting time: 125.5 days in the early phase, 9 days in the rapid scale-up phase, and 15 days in the recent cohort (Chi-square = 120, P<0.001).

Patients with WHO stage III disease constituted 49.2%, stage II 19.4%, stage I 18.2%, stage IV 13.3% at the time of presentation to care. The recent cohort had the largest proportion of patients in stage II (33.9%) and the smallest in stage IV. Patients in the oldest cohort were more likely to be in advanced clinical stage compared with those in the recent cohort (adjusted OR = 4.03, 95% CI 3.07–5.27). Similarly, men compared to women (adjusted OR = 1.78, 95%CI 1.47–2.16), those aged 45 years and above versus younger ones (adjusted OR = 2.04, 95%CI 1.48–2.82) as well had higher risk of being in advanced WHO clinical stage. Also, being divorced, widowed or separated as compared with being married was associated with higher risk of being in advanced clinical stage. Table 2 shows factors associated with being in advanced clinical stage at presentation.

**Table 2:** Logistic regression analysis of factors associated with being in advanced WHO clinical stage (WHO stage III&IV) for all patients, Arba Minch Hospital, 2010.

Predicator variable	Unadjusted OR (95% CI)	P-value	Adjusted OR (95% CI)	P-value
Phase of enrollment				
Recent phase	1.00		1.00	
Rapid scale-up phase	2.23 (1.82–2.72)	<0.001	2.12 (1.73–2.61)	<0.001
Early phase	4.16 (3.21–5.39)	<0.001	4.03 (3.07–5.27)	<0.001
Marital status				
Married	1.00		1.00	
Unmarried	1.23 (0.96–1.58)	>0.1	1.15 (0.88–1.51)	>0.05
Divorced, widowed or separated	1.34 (1.15–1.71)	<0.01	1.38 (1.11–1.71)	<0.01
Sex				
Female	1.00		1.00	
Male	1.81 (1.51–2.16)	<0.001	1.78 (1.47–2.16)	<0.001
Age in years				
15–44	1.00		1.00	
45+	2.34 (1.73–3.17)	<0.001	2.04 (1.48–2.82)	<0.001

The 1428 treatment naïve patients who started ART contributed 2422.4 person years of observation (PYO). More than a half were women (53.6%), the mean age was 34 (SD  =  +/−8.8) years, over a half (53.2%) were married, and most (84.7%) were from urban areas. Baseline CD4 was available for 1037 patients (median CD4 value 156 cells/mm^3^ (IQR = 81, 237), and 649 (62.5%) patients had CD4 values less than 200 cells/mm^3^. In 1288 patients with baseline TLC, the median value was 1300 cells/mm^3^ (IQR = 900, 1900).

Combined CD4 and clinical stage accounted for 40.5% (578 patients) of the indications to start ART followed by TLC and clinical stage in 29.7% (424 Patients), CD4 only in 26.2% (374 patients), and clinical stage only in 3.6% (52 patients) of patients.

Table 3 summarizes the clinical stage at the beginning of ART, and shows that patients coming to the hospital now start treatment earlier.

**Table 3:** WHO clinical stage at start of antiretroviral treatment at Arba Minch Hospital, 2010.

Phase of ART initiation	WHO Stage		Total, count (%)
	I and II, count (%)	III and IV, count (%)	
Early	14 (6.3)	209 (93.7)	223 (100)
Rapid-scale up	135 (17.3)	646 (82.7)	781 (100)
Recent	148 (34.9)	276 (65.1)	424 (100)
Total	297 (20.8)	1131 (79.2)	1428 (100)

Chi–square for trend (X^2^ = 83.3; df  = 1; P<0.001).

As first line regimen, 606 patients (42.4%) received d4t/3TC/NVP and 564 patients (39.5%) received d4t/3TC/EFV. 136 patients (9.5%) received AZT/3TC/EFV, 110 patients (7.7%) received AZT/3TC/NVP and the remaining 12 patients (0.8%) received a TDF containing regimen.

### Follow-up results

#### Pre-ART follow-up

Overall, 2191 patients contributed 777.8 person-years of observation (PYO). The median time between enrolment and pre-ART outcome was 31 days (IQR, 5–130 days). The pre-ART mortality rate was 13.1 per 100 PYO (102 deaths/777.8 PYO), with the highest mortality being during the early phase (84 deaths/323.9 PYO = 25.9 per 100 PYO) and the lowest rate (4 deaths/287.8 PYO = 1.4/100 PYO) during the rapid scale-up phase. Some increment in mortality was seen in the recent cohort (14 deaths/166 PYO = 8.4 per 100 PYO).

**Figure 2:**
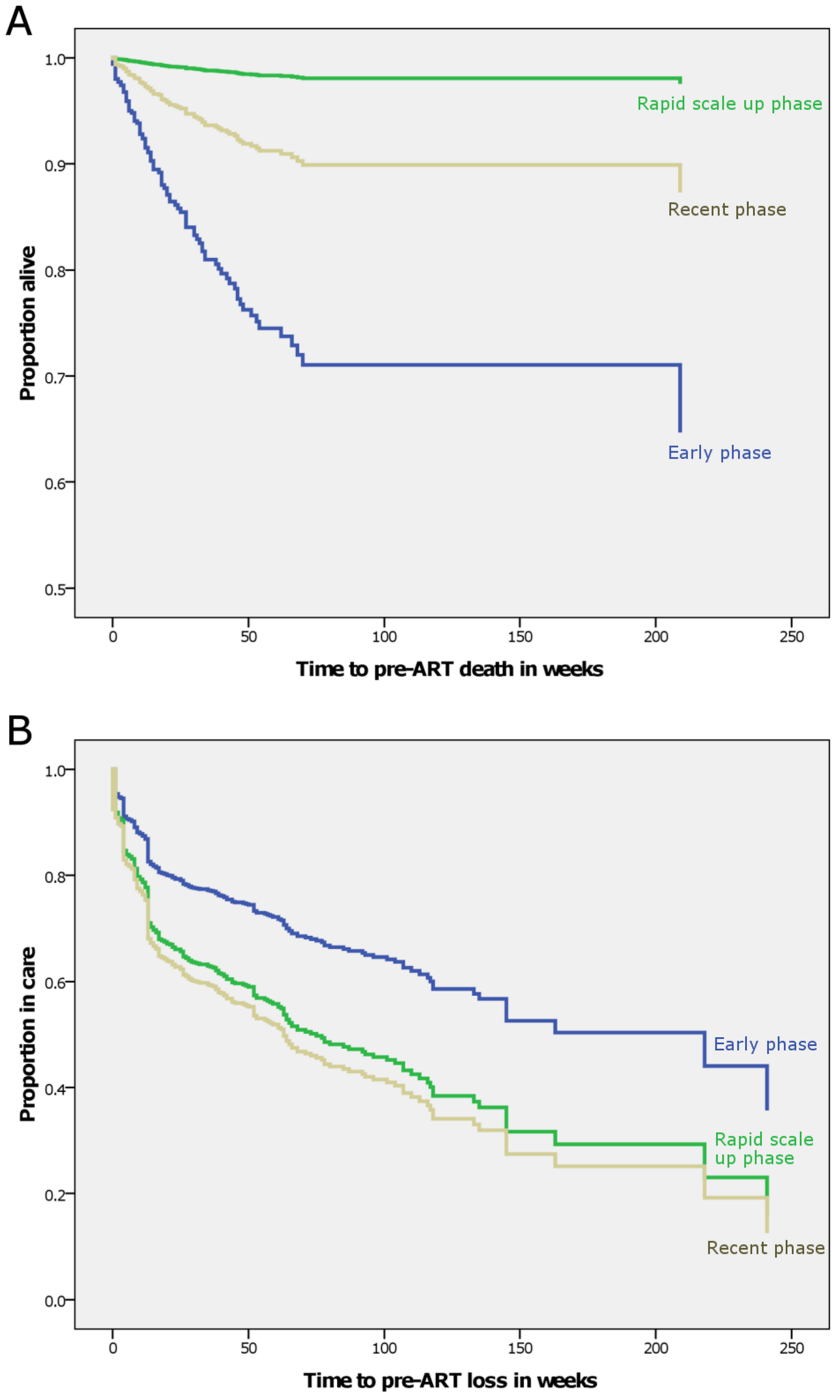
Survival curve according to Cox regression analysis after adjusting for disease stage, age and place of residence, Arba Minch Hospital, 2010 . At about 48 weeks of pre-ART follow-up, about 25% of patients in the ‘early phase’ were not alive (See 2A). On the other hand, over 95% of those in the ‘rapid scale-up’ phase were alive at 48 weeks. Pre-ART loss to follow-up was less in the ‘early phase’ (See 2B).

In adjusted Cox- regression analyses controlling for WHO stage, TLC, and Hgb, patients enrolled during the early phase were more likely to die compared with those in the rapid scale-up phase (adjusted HR = 2.45, 95% CI = 1.31,4.61). Also, patients in advanced WHO stage compared to those in less advanced stage (adjusted HR = 2.8, 95% CI = 1.6, 4.8) and those having TLC less than or equal to the median value were more likely to die (adjusted HR = 1.6, 95% CI = 1.01, 2.42) during the pre-ART period. Figure 2A shows survival according to adjusted Cox-regression analyses.

A quarter of the patients were lost to follow-up. Being in less advanced WHO clinical stage (adjusted HR = 1.9, 95% CI = 1.6, 2.2), being in the recent cohort compared to being in early phase (adjusted HR = 2.0, 95% CI = 1.6, 2.6), and being a rural resident (adjusted HR = 1.8, 95% CI = 1.5, 2.2) were independent predictors of loss to follow-up. Age, sex and marital status were not associated with being lost to follow-up. Figure 2B shows adjusted time to pre-ART loss for the three time periods according to Cox-regression analysis.

#### On-ART follow-up

The median follow-up time was 17.7 (IQR 6.2, 31.5) months. At the end of the follow-up, 901 (63.1%) patients were alive and under follow-up, 191 (13.4%) were lost to follow up, 171 (12%) were transferred out, 161 (11.3%) had died, and four (0.3%) patients had stopped treatment (Figure 1).

The loss to follow up rate was 8.2 per 100 PYO (191 patients per 2315 PYO). Of these 191 patients, we were able to trace 143 patients (response rate of 143/191 (75%)). Of the 143 patients, 58 (40.6%) patients had died, 29 (20.3%) were under follow up at another health institution, 20 (13.9%) had stopped treatment and were alive, 14 (9.8%) had left the region, 13 (9.1%) used traditional treatment, and in 9 (6.3%) patients the outcome remains unknown. We also traced and found the four patients who stopped treatment. Of these, one later restarted treatment, one had died and two patients were alive but did not restart treatment.

Table 4 shows risk rates and hazard ratios of the 191 patients registered as lost to follow up. The results show that patients starting treatment during the rapid scale-up (HR [95%CI]  = 2 [1.1, 2.9]) and recent phase (HR [95%CI]  = 2.1[1.2, 3.8]) had increased risk of loss to follow-up. Patients living in towns, and those who had drug regimen change were less likely to default treatment.

**Table 4:** Hazard ratio (HR) for lost to follow up among patients on antiretroviral treatment at Arba Minch Hospital, 2010.

Category	Person years of observation (PYO)	Number of patients lost to follow up	Lost to follow up rate (per 100 PYO)	Unadjusted HR[95%CI]	Adjusted HR[95%CI]
Age in years					
< = 45	2097.2	175	8.3	1	1
>45	217.8	16	7.3	0.9 [0.5,1.5]	0.9 [0.5,1.5]
Address					
Rural	233.4	47	20.1	1	1
Urban	2081.6	144	6.9	0.3 [0.2,0.5]	0.5 [0.3,0.6]
Gender					
Female	1253.1	104	8.3	1	1
Male	1061.9	87	8.2	0.9 [0.7,1.3]	0.9 [0.7,1.3]
Phase of ART initiation					
Early	584.6	17	3	1	1
Rapid scale-up	1448.4	125	8.6	2.9 [1.8,5.1]	2 [1.1,2.9]
Recent	282	49	17.4	5.9 [3.5,10.6]	2.1 [1.2,3.8]
WHO stage					
I and II	392.8	32	8.1	1	1
III and IV	1922.2	159	8.3	1.1 [0.7,1.5]	1.2 [0.9,1.9]
Change of drug regimen					
Yes	598.8	15	2.5	0.2 [0.1,0.4]	0.4 [0.2,0.6]
No	1716.2	176	10.3	1	1

CI =  Confidence Interval, HR =  Hazard ratio, Person Years of Observation.

**Figure 3:**
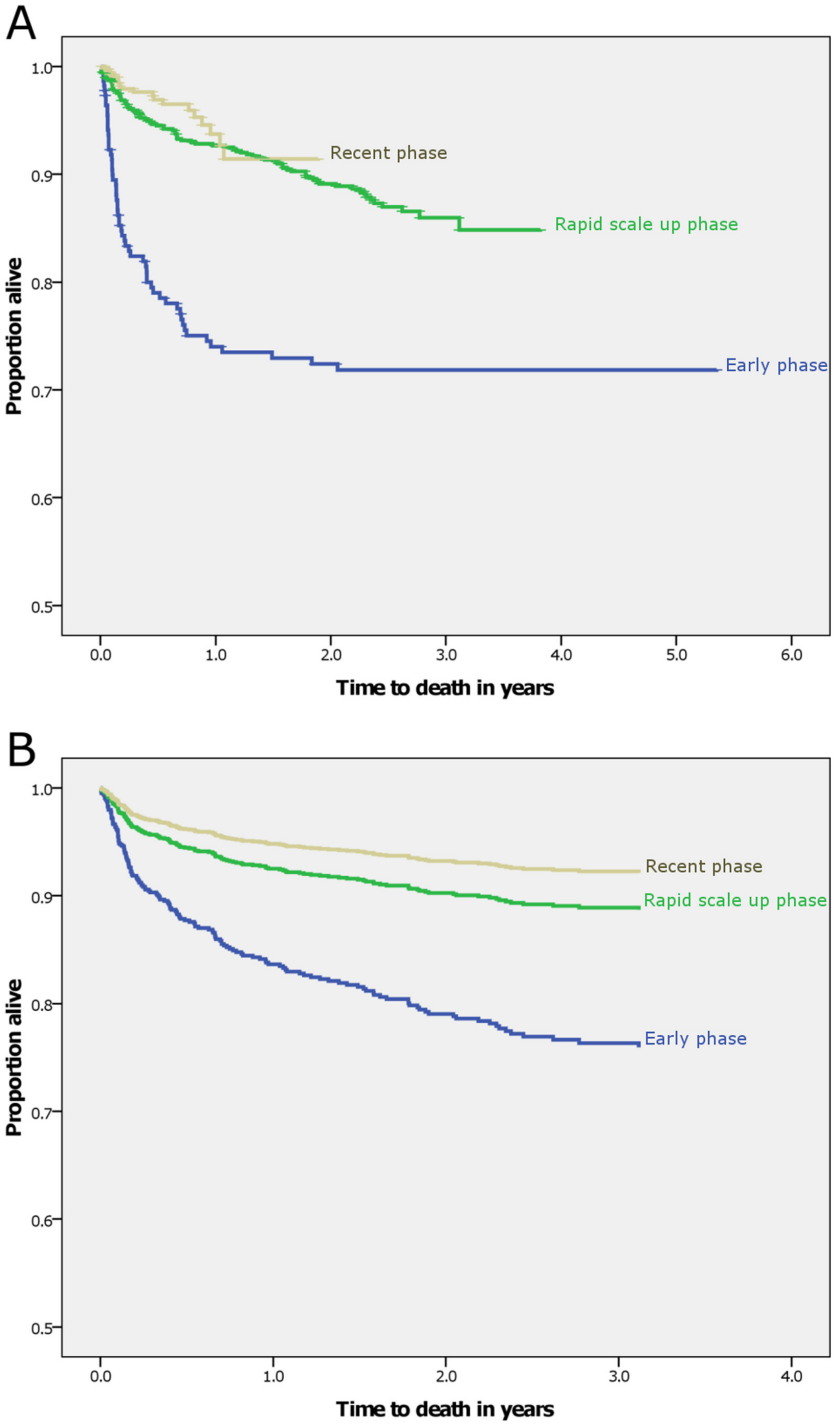
Kaplan –Meier (3 A) and Cox-proportional hazard's (3 B) survival plots according to the three periods of ART initiation, Arba Minch Hospital, 2010.

The overall mortality rate was 9.1 per100 PYO (220 deaths per 2422.4 PYO). The survival of patients on ART improved during the study period (Figure 3, Tables 4 and 5) and is associated with improvement in patients starting treatment earlier (Table 3).

**Table 5:** Hazard ratio (HR) for death among patients on antiretroviral treatment at Arba Minch Hospital, 2010.

Category	Person years of observation (PYO)	Number of deaths	Death rate Per 100 PYO	Unadjusted HR[95%CI]	Adjusted HR[95%CI]
Age in years					
< = 45	2203.2	186	8.4	1	
>45	219.2	34	15.5	1.8 [1.3,2.6]	1.7 [1.2,2.4]
Address					
Rural	234.2	27	11.5	1	1
Urban	2188.2	193	8.8	0.8 [0.5,1.2]	0.9 [0.6,1.5]
Gender					
Female	1319.1	94	7.1	1	
Male	1103.3	126	11.4	1.6 [1.2,2.1]	1.4 [1.1,1.8]
Phase of ART initiation					
Early	608.4	62	10.2	1	1
Rapid scale-up	1522.3	130	8.5	0.8 [0.6,1.1]	0.5 [0.4,0.7]
Recent	291.7	28	9.6	0.9 [0.6,1.5]	0.4 [0.2,0.6]
WHO stage					
I and II	412.1	17	4.1	1	1
III and IV	2010.3	203	10.1	2.4 [1.5,4.1]	2.4 [1.5,4]
Change of drug regimen					
No	1815.6	186	10.2	1	
Yes	606.8	34	5.6	0.5 [0.4–0.8]	0.5 [0.4,0.8]

CI =  Confidence Interval, HR =  Hazard ratio, PYO =  Person Years of Observation.

Higher mortality was associated with advanced clinical stage at start of treatment (HR [95%CI]  = 2.4 [1.5, 4]), age over 45 years at presentation (HR [95%CI]  = 1.7 [1.2, 2.4]) and men had higher mortality rates than women (HR [95%CI]  = 1.4 [1.1, 1.8]) (Table 5).

Mortality varied with baseline CD4 count: 67 (10.4%) patients with CD4 counts less than 200 cells/mm^3^ died [7.9 deaths per 100 PYO] compared with 13 (2.7%) deaths in patients with CD4 count 200–350 cells/mm^3^ [2.7 deaths per 100 PYO. From the 391 patients with no initial CD4 count, 79 (20.2%) died [8.6 deaths per 100 PYO].

## Discussion

Patients now present themselves with less advanced disease than during the early years, and our data suggest that earlier treatment start is the main reason for improved survival. However, patients in the recent cohort, rural residents and those in less advanced disease stage were more likely to default before starting treatment. This is in addition to the already high on-ART loss to follow-up rate, which we confirmed in this long-term follow-up.

The high pre-ART loss to follow-up rate is a worrying phenomenon. Of particular concern is the higher loss rate among patients with less advanced clinical stage who are likely to be engaged in risky sexual practices. Part of the underlying reasons for the high rate of loss among patients with less advanced disease stage could be lack of means for engaging them in care. Such patients often do not need treatment and prophylaxis for opportunistic infections. Prophylactic interventions such as isoniazid preventive therapy (IPT) are not widely implemented in the study setting, as is elsewhere in Ethiopia. Also, there was no mechanism for pre-ART patient tracing.

There is limited data on trends in immune status at presentation among patients followed in settings with limited resources. In a cohort followed in a well-resourced setting in the US, there was no improvement in immune status at presentation after 16 years of follow-up. [12] Similar to our finding, however, there was decrease in time from HIV diagnosis to presentation for care.

High rates of pre-ART mortality and loss to care were reported from South Africa, Uganda, and Cambodia. [13], [14], [15], [16], [17], [18] In a South African cohort, a pre-treatment mortality rate of 33.3 per 100 PYO was reported. Pre-treatment and early treatment deaths accounted for 87% of all deaths. [20] Among Ugandan cohort of HIV patients, pre-ART mortality rate of 27 per 100 PYO was reported. [13] An overall pre-ART mortality rate of 13.1 per 100 PYO in our cohort is much lower than the reports from the aforementioned studies. However, when stratified by year of enrolment, a mortality rate of 25.9 per 100 PYO among our oldest cohort is comparable to other studies.

There are explanations for the higher mortality rate among the oldest cohort. Between January 2003 and August 2003, patients were followed without ART as there was none in the country. Ethiopia did not have a policy on ARV drug use until July 2002. [19] Even when ART was made available, it took some time until decision was made as to which patients would be prioritized. Also, the improved clinical mentoring approach used by JHU-TSEHAI coupled with the improved experience gained by the clinicians could have contributed to the improved patient outcomes at later dates.

Pre-ART loss to follow-up is a common but less clearly defined challenge in settings with limited resources. [13], [14], [15], [16] At a partly private clinic in Durban, South Africa, 16.4% of the patients were lost within three months' of follow-up before ART initiation. [15] In their report, having low CD4 count was associated with being lost to follow-up, and over a third of them were found to be dead. The higher loss rate among patients with low CD4 count is in sharp contrast to our finding of higher loss rate among patients with better immune status. This could be explained by the difference in the approach used by the two settings.

In Uganda, inadequate post-test counselling and competition from holistic and less stigmatizing traditional/spiritual healers were cited as the main reasons for pre-ART loss. [20] Also, transportation costs, long waiting time, lack of incentives to seek pre-ART care by healthy looking patients and gender inequalities were mentioned as some of the perceived reasons for the high loss.

Our on-ART loss to follow up rates are high, but comparable to findings from 10 African and Asian countries reported in a recent meta-analysis [21]. About 40% of the patients lost to follow up were later found to have died, and this figure is also comparable with meta analysis reported by Brinkhof and colleagues [21].

In our study about 20% of those lost to follow up were found to be receiving care in another health institution, suggesting that self-transfer of patients to ART centres of their preference is common. There is a need for strengthening communication between health institutions.

The on-ART mortality rate (9.1 per 100 PYO) in our study is higher than rates from China (5.9 per100 PYO) [22], but similar to a recent review from four African countries (mortality rate 8 per 100 PYO)[23]. A Ugandan study showed lower mortality rates (3.5 per 100 PYO) [24], probably because more Ugandan patients were in WHO stage II (63% compared with 43% in our study).

As expected, we found that patients with advanced clinical disease or low CD4 count have higher risk of mortality. Possible causes of higher mortality rates among the older patients could be because older patients respond poorly to ART and experience more rapid clinical progression. Older patients are also at a higher risk of complications such as cancer and cardiovascular disease because of the combined effect of ageing, HIV infection and antiretroviral treatment [25]. However, there is limited data from Africa trying to explain higher mortality rates among the older people. Although we controlled for possible confounders such as WHO stage, age, CD4, TLC and home place, men had higher risk of death, as also reported from four African countries [23] and cohort studies in Europe and North America [26]], but not from Spain[27]. Possible reasons for higher mortality among males needs further study.

In conclusion, patients visiting Arba Minch Hospital for HIV treatment have started to present at less advanced disease stages. This was accompanied by a decline in patient mortality rate. However, high rate of pre-ART loss to follow up especially among well-looking and rural patients appears to be a growing challenge. This suggests the need for strengthening the pre-ART phase of HIV care. Also, we documented high rate of loss to follow up of patients on ART. Thus, there is an urgent need to enhance the community tracing of patients defaulting. The country now uses adherence supporters (most of whom are PLHIV) as adherence counsellors and defaulter tracers.

Health care workers in similar settings should pay more attention to clients who are likely to default during the pre-ART phase. More targeted counselling and follow-up is needed. Also, existing interventions such as IPT and management of other opportunistic infections could be used as incentives to engage patients in care. [28], [29] There is a need for clear policy on pre-ART patient care including the intensity of follow up and monitoring and evaluation tools. Definitions for pre-ART loss should be standardized. The human resource implications, cost and cost-effectiveness of instituting pre-ART care package should be studied.
